# Interferon Control of the Sterol Metabolic Network: Bidirectional Molecular Circuitry-Mediating Host Protection

**DOI:** 10.3389/fimmu.2016.00634

**Published:** 2016-12-23

**Authors:** Kevin A. Robertson, Peter Ghazal

**Affiliations:** ^1^Division of Infection and Pathway Medicine, University of Edinburgh, Edinburgh, UK

**Keywords:** cholesterol, sterol, interferon, metabolism, miRNA, oxysterol, 25-hydroxycholesterol, miR-342-5p

## Abstract

The sterol metabolic network is emerging center stage in inflammation and immunity. Historically, observational clinical studies show that hypocholesterolemia is a common side effect of interferon (IFN) treatment. More recently, comprehensive systems-wide investigations of the macrophage IFN response reveal a direct molecular link between cholesterol metabolism and infection. Upon infection, flux through the sterol metabolic network is acutely moderated by the IFN response at multiple regulatory levels. The precise mechanisms by which IFN regulates the mevalonate-sterol pathway—the spine of the network—are beginning to be unraveled. In this review, we discuss our current understanding of the multifactorial mechanisms by which IFN regulates the sterol pathway. We also consider bidirectional communications resulting in sterol metabolism regulation of immunity. Finally, we deliberate on how this fundamental interaction functions as an integral element of host protective responses to infection and harmful inflammation.

## Introduction

Immunity depends on and employs metabolic pathways for its function. Our knowledge of the molecular and functional mechanisms for this coupling has grown dramatically in recent years and it is now accepted that a remodeling of glycololytic, lipid biosynthetic, and associated homeostatic molecular “circuitry” is an integral component of innate and adaptive immune responses (1–3). In particular, multiple immune-mediated mechanisms for the transcriptional, posttranscriptional, translational, and posttranslational regulation of lipid biosynthesis, storage, influx, and efflux in immune cells have been described (4–7). Broadly, with some notable exceptions, these mechanisms have been defined *in vitro* in specific cell types (e.g., macrophages) and their general significance and relative importance *in vivo* have yet to be fully characterized.

**Figure 1:**
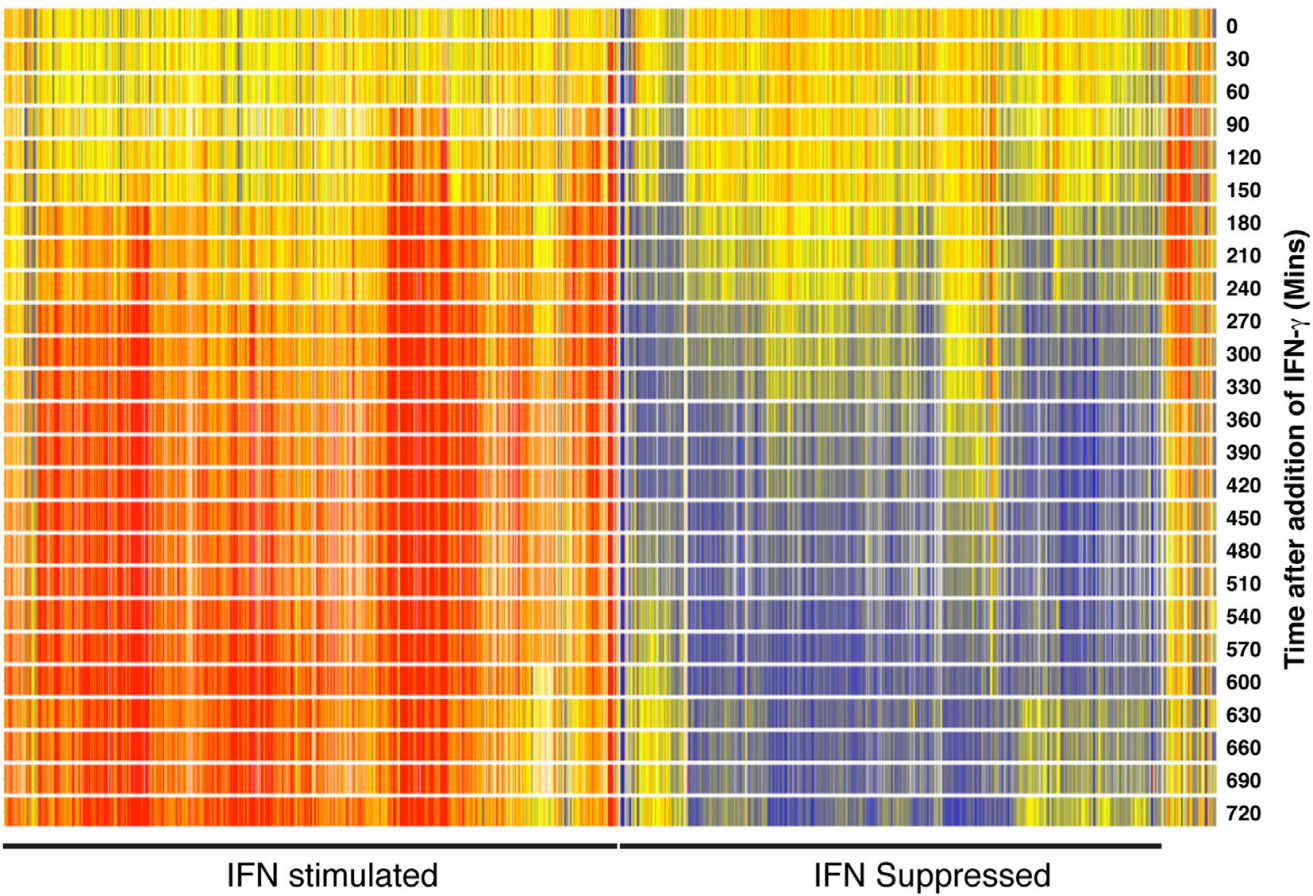
**Heat map showing 1,048 genes significantly increased or decreased in expression after interferon (IFN) simulation of macrophages**. Bone marrow-derived macrophages were mock treated or treated with 10 U/ml IFN and then sampled at 30-min intervals for a period of 12 h. Total RNA was then labeled and hybridized to Mouse Agilent V2 (G4121A) microarrays. Gene expression is shown as a pseudo-color—blue, decrease; red, increase. Explorative and statistical analyses were undertaken as previously described (4). Data are available for download from the NCBI gene expression omnibus (https://www.ncbi.nlm.nih.gov/geo/) (series GSE42504).

Immediately after infection, the ligation of cellular pattern-recognition receptors by, for example, dsRNA leads to an induction of NFkB, ATF2/c-jun, and interferon regulatory factor 3 (IRF3), a rapid upregulation of IFNα/β gene expression and secretion of type I IFNs by cells. The autocrine/paracrine binding of IFNα/β or IFN-γ (from activated NK and T cells) to type I or type II IFN receptors, respectively, leads to the activation of JAK/STAT signaling pathways and rapid alterations in the abundance of hundreds of transcripts in the cell. These IFN-stimulated changes reflect an acute re-programing of the cell to resist infection and limit cellular damage. Figure 1 shows a high-resolution temporal (every 30 min for the first 12 h) analysis of genome-wide alterations in gene expression upon IFN-γ activation of bone marrow-derived macrophages. Importantly, alongside many IFN-stimulated genes, this data reveal an equivalent number of transcripts are significantly suppressed by IFN.

While interferon (IFN)-stimulated genes (ISG) such as *NOS2, OAS2, MX2*, and *IFITM3* have intensively investigated antiviral or antibacterial effects, IFN downregulated transcripts have received relatively little attention (8–11). Notably, a statistical over-representation analysis of the IFN suppressed genes presented in Figure 1 identified the sterol metabolic network as a significantly over-represented component of this dataset. Importantly, consequent mechanistic studies demonstrated that a suppression of sterol biosynthesis is an integral component of the innate immune response to infection (4). This work raised several significant questions about the coupling of sterol metabolism to immunity. In particular, what are the molecular mechanisms by which IFN mediates a downregulation of the sterol biosynthesis pathway and how does the suppression of sterol biosynthesis benefit the infected host? Recent studies are beginning to answer some of these questions.

Here, we first discuss early clinical work showing iatrogenic effects of IFN on sterol metabolism. Next, with an emphasis on molecular oxysterol and miRNA-mediated mechanisms, we consider what is known about how IFN regulates sterol metabolism. Overall, we advance the notion that the mevalonate–sterol pathway is an effector arm of immunity and highlight how this response helps the host limit excessive inflammation and resist infection.

## Hypocholesterolemic Effects of IFN Treatment in Humans

Although interest in IFN-mediated regulation of the sterol pathway has increased dramatically in recent years, IFN-induced alterations in cholesterol in humans have been reported for several decades (Table 1). In 1979, Baillie and Orr reported that acute viral infections are regularly associated with reductions in systemic cholesterol in patients (12). Subsequently, Cantell et al. (13) showed that the administration of partly purified human leukocyte IFN to volunteers led to a 20% drop in high-density lipoprotein (HDL), a transient declining trend in total cholesterol and put forward the first proposal that viral infections elicit a drop in cholesterol *via* the induction of IFN (13). Table 1 presents a chronological summary of wide-ranging studies in which natural and recombinant type I and type II IFNs have been administered to volunteers or patients with cancer, multiple sclerosis, human papilloma, or hepatitis C virus (HCV) infections. In all studies, despite differences in the preparation of IFN used, a drop in circulating total cholesterol and/or HDL was observed. Arguably, the strongest clinical evidence comes from prospective double blind studies such as those reported by Rosenzweig et al. (14) and Borden et al. (15). The former utilized a double blind analysis to demonstrate a dose-dependent effect of IFN administration on plasma cholesterol (14). The latter employed a prospective double blind placebo-controlled analysis of IFN treatment in renal carcinoma patients, demonstrating a significant decrease in mean plasma total cholesterol (15). It is worth noting that Rosenzweig et al. (14) also showed that the effects of IFN were not permanent and that after cessation of treatment circulating cholesterol levels returned to normal in patients (14). In subsequent metabolic tracer experiments, the primary effect of IFN was shown to occur *via* a modulation of cholesterol synthesis (16).

**Table 1:** **Representative clinical studies reporting decreases in cholesterol following treatment with type 1 or 2 IFN**.

IFN type	Year	Treatment	Disease context	Observation	Reference
Partly purified human leukocyte IFN	1980	1× healthy male volunteer: 10× daily SC injections of 3 × 10^6^ IU. Two further volunteers: 1× SC of 3 × 10^6^ IU, then 3 × 1.5 × 10^6^ IU on consecutive days	Healthy volunteer	Drop in high-density lipoprotein (HDL) cholesterol in all volunteers 7 days after treatment	(13)
Human leukocyte IFN	1981	3 × 10^6^ IU IFN IM daily for 1 week. 6 × healthy male	Healthy volunteer	Total and HDL plasma cholesterol decreased in all 6 subjects	(17)
Human IFN-α prepared from buffy coat leukocytes rIFN-αA (Hoffman–LaRoche Inc., Nutly, NJ, USA)	1984	Daily IM injection of 3 × 10^6^–9 × 10^6^ U of (A) over 28–57 days Daily IM injection of 3 × 10^6^–5.4 × 10^7^ U of (B) for 15 days	Cancer	Significant decrease in HDL and total cholesterol	(18)
rIFN-β_ser_ (modified rIFN-β: Ser_17_ substituted for cysteine)	1985	Escalating dose regime: IM and IV injection from 1 × 10^6^ to 4 × 10^8^ U, twice weekly	Cancer	Decrease in serum cholesterol	(19)
rIFN-α2	1986	3 × 10^7^ U/m^2^ IV for 5 days consecutively every 3 weeks	Cancer	Significant decrease in plasma cholesterol. Effect specific to low-density lipoprotein (LDL) and HDL. VLDL or triglycerides unchanged	(20)
rIFN-β_ser_	1987	Patients randomly assigned to 1 of 2 dose regimens. 4.5 × 10^6^ U (3 males and 7 females) or 9 × 10^7^ U (8 males and 3 females) of IFN-β_ser_ IV daily in a double blind manner for 10 days followed by 11 days off	Cancer	Significant dose-dependent decrease in mean plasma total cholesterol and LDL concentrations (24–36 h after initiation of treatment). Approx. 25% reduction in plasma cholesterol concentration after 10 days of treatment	(14)
IFN-α-n1 (Wellferon—highly purified combination of natural human IFNα from lymphoblastoid cells)	1988	9× men received IM treatment	Refractory condylomata acuminata	All patients had significant decrease in HDL cholesterol levels. Total cholesterol decreased—change not significant	(21)
rIFN-β_ser_	1990	Randomized, double-blind trial of two doses of IFN-β_ser_ (4.5 × 10^6^ and 9 × 10^7^ U). IV injections daily for 10 days with 11 days rest before treatment reinitiated	Cancer	Statistically significant change in cholesterol	(15)
rIFN-γ	1990	29 patients treated IV at doses escalating from 2 × 10^5^ to 10^8^ IU/m^2^ in 9 successive steps (at least 3 patients/step). Injections of rIFN gamma were repeated every 72 h for 15 days	Cancer	Hypocholesterolemia observed in 18 patients	(22)
rIFN-β_ser_	1992	4.5 × 10^6^ U daily IV for 5 weeks to normal and hypercholesteremic patients	Hypercholesteremia	Significant 15% reduction of total cholesterol in normal and hypercholesterolemic subjects. IFN induced significant reductions in LDL cholesterol of 25% in normal subjects and of 40% in hypercholesterolemic subjects. Significant decreases in LDL apoB observed only in the normal group	(16)
rIFN-α2b	1995	44 patients were treated with human recombinant interferon (IFN)-alpha 2b (3 × 10^6^ U 3× per week for up to 12 months). 8 control patients	Hepatitis C virus (HCV)	Blood lipids evaluated after 3, 30, and 90 days of treatment. HDL, cholesterol, apolipoprotein A-I, and HDL3 decreased within 4 weeks of starting IFN treatment	(23)
rIFN-α2a	1997	39 patients: recombinant IFN alpha-2a (9 × 10^6^ U/day) administered IM for 2 weeks, and then 3× a week for 6 months	HCV	Serum cholesterol concentration significantly decreased 1 week after start of IFN administration. 67% of reduction attributable to HDL-cholesterol	(24)
rIFN-α2b (Intron A, Schering–Plough, Kenilworth, NJ, USA)	1998	36 patients received therapy with recombinant IFN-α2b for 6 months; 34 patients received 5 × 10^6^ U and 2 patients 6 × 10^6^ U, 3× a week	HCV	Reduction in HDL-cholesterol and apoA1 levels. Total, LDL, and lipoprotein(a) levels unchanged during treatment	(25)
rIFN-β (Frone, Serono, Madrid, Spain)	2000	IFN-β SC (6 × 10^6^ U) 3× a week for 6 months	HCV	Cholesterol concentration decreased slightly in HDL subfractions	(26)
rIFNβ-1a (Avonex; Biogen Idec, Inc., Cambridge, MA, USA) rIFNβ1b (Betaferon—cys_17_ replaced by ser_17_, lacks met_1_ and carbohydrate moieties—Schering, Berlin, Germany) rIFNβ1a (Rebif, Ares-Serono, Geneva Switzerland)	2004	95 patients: 6 × 10^6^ U/week IM and SC IFNβ1a (Avonex) 92 patients: 8 × 10^6^ U IFNβ1b every other day SC 41 patients: 22 µg 3× SC/week. IFNβ1a (Rebif) 25 patients: 3× SC/week 4 µg IFNβ1a (Rebif)	MS	Highly significant sustained decrease (−8%) in mean cholesterol level in plasma of IFN-treated MS patients	(27)
rIFNβ-1a (Avonex; Biogen Idec, Inc., Cambridge, MA, USA)	2006	255 patients were included in the study	MS	Decrease in blood cholesterol	(28)
Peg-rIFN	2016	520 patients treated with pegIFN or combination of IFN-free direct acting antivirals (DAA)	HCV	IFN-based therapy decreased total circulating cholesterol, while IFN-free DAA increased cholesterol levels	(29)

In summary, the induction of hypocholesteremia by IFN has been recorded clinically for many years *via* an analysis of total cholesterol, HDL, or LDL in the circulation. Despite this recognition, physiological roles related to human health and underpinning this observation have not been further investigated.

## The Mevalonate-Sterol Pathway is an Intrinsic Component of the IFN Response to Infection

Alongside, clinical studies demonstrating exogenously administered IFN can regulate sterol metabolism, a number of groups have also associated cholesterol regulation with IFN responses in experimental animal studies. In 1984, Kuo et al. showed that IFN-inducing agents significantly reduced cholesterol deposits in the aortas of rabbits fed a pro-atherogenic diet (30). Further, in 1987, Pereira et al. showed that a hypercholesteremic diet resulted in an increased susceptibility to murine hepatitis virus in A/J mice—a result in part due to a decreased response to IFN and reduced antiviral state (31). A key question in this context is: *what benefit to the host is conferred by the IFN regulation of sterol metabolism?* While studies prior to 2011 showed that toll-like receptor 3 (TLR3) or TLR4 ligation results in an IFN-independent inhibition of cholesterol efflux from the cell, little was known at this point about how IFN signaling directly influences cholesterol homeostasis and the physiological purpose this could serve (32). In 2011, Blanc et al. demonstrated that viral infection or treatment of macrophages with type I or II IFN results in a coordinate, negative regulation of the entire sterol biosynthesis pathway and that inflammatory cytokines such as TNF, IL-6, and IL1β are incapable of eliciting a similar effect. This study further showed that the regulation of the sterol pathway by IFN is, at least partly, due to a reduction in SREBF2 transcription and SREBP2 abundance and that this event is an integral component of the cell-intrinsic antiviral response (4). Notably, a recent study highlighted the interdependent reciprocal nature of the molecular circuitry coupling IFN and sterol metabolism. In 2015, York et al. described a STING-dependent recognition of decreased flux through the sterol biosynthetic pathway leading to positive feedback that enhances the type I IFN response and antiviral gene expression in the context of gammaherpesvirus infection (33). The implications of this data are discussed later in this review.

## Functional Roles for the Mevalonate-Sterol Pathway in Governing Adaptive Immune Responses

Beyond the intracellular and/or cell-intrinsic environment, IFN-mediated regulation of sterol metabolism has the potential to influence many aspects of immunity. The functions that the sterol metabolic network plays in a wide range of adaptive immune responses have recently been reviewed (3). These include: an absolute requirement for SREBP2 functionality during activated T lymphocyte clonal expansion, a requirement for flux through the sterol biosynthesis pathway during the activation of T regulatory cell function, the observation that a hypercholesteremia can alter the balance of the Treg and T effector cells, and the induction of lymphocyte hyper-proliferation due to impaired cholesterol efflux (34–38). Cholesterol is also indispensable in the formation of lipid raft microdomains—crucial to the assembly of cell surface signaling molecules such as the T and B cell receptors—and has recently been identified as a critical allosteric regulator of TCR priming (39, 40). Notably, cholesterol is not the only output of the sterol biosynthesis pathway on which cells depend. For example, prenylation of the Ras family of GTPases by the side branch of the mevalonate pathway is integral to the control of T cell differentiation, proliferation, and cytokine production [reviewed in Ref. (41)]. Further, sterol pathway intermediates have also been identified as endogenous ligands for the transcription factor RORγt. RORγt is required for the differentiation of naïve CD4+ T lymphocytes into T_H_17 cells, a subset of lymphocytes associated with a range of autoimmune diseases and mediating protective immune responses to pathogens such as *Klebsiella pneumoniae, Bordetella pertussis, Mycobacterium tuberculosis*, and *Candida albicans* (42–45). Santori and colleagues identified sterol pathway intermediates generated downstream of lanosterol and above zymosterol as natural ligands of RORγt, while Hu et al. suggested that desmosterol (downstream of zymosterol and recently shown to be negatively regulated by IFN) is a potential endogenous ligand for this transcription factor (7, 46). Through the utilization of chemical library screening studies, the oxysterol 7β,26-dihydroxycholesterol (synthesized from 7β-hydroxycholesterol, a metabolite immediately downstream of cholesterol) has also been identified as a potent ligand of RORγt (47). In summary, the sterol metabolic network is increasingly viewed as integral to the activation and differentiation of T lymphocytes. More work, however, is required to better understand the precise mechanisms by which the network and/or specific metabolites function in these processes.

In B lymphocytes, IFN regulation of sterol metabolism may lead to alterations in lipid raft cholesterol composition and, in doing so, affect antigen processing/presentation (48) and B-cell receptor signaling (48–50). Notably, significant roles for the inflammatory sterol pathway product 25-hydroxycholesterol and 7α,25-dihydroxycholesterol in class-switching and the chemoattraction of B lymphocytes to germinal centers have recently been described and these will be discussed in detail later.

## Benefits of IFN-Mediated Sterol Regulation During Infection

An IFN-mediated suppression of sterol metabolism has the potential to directly curtail the replication of microorganisms in the host. Pathogens with a dependency on the host sterol metabolic network include HCV, human immunodeficiency virus (HIV), Ebola, the Herpesvirus family [HCMV, murine cytomegalovirus (MCMV), herpes simplex virus type (HSV1) MHV-68, and varicella zoster virus (VZV)], Influenza A virus (IAV), *Listeria monocytogenes*, and *M. tuberculosis* (51–57). Importantly, the requirements of these organisms on the system vary dramatically. For example, lipid rafts play an integral role in the entry, assembly, and release of a wide range of unrelated viruses (enveloped and non-enveloped) such as HIV1, Ebola, Influenza A, and Echovirus 1 [reviewed here in Ref. (58)]. In contrast, HCMV uses cholesterol for envelopment and limiting the availability of intracellular cholesterol levels has been shown to restrict infectivity of this virus (59). The replication of several viruses also requires prenylation of host and/or virus proteins. For example, hepatitis D virus requires prenylation of its large delta antigen for optimal virion morphogenesis, HCV requires the geranylgeranylated host protein FBL2 for replication, and respiratory syncytial virus (RSV) F glycoprotein binds to the prenylated host protein RHOA enabling membrane fusion (60–62). In the context of bacterial infection, Listeriolysin O, the major virulence factor of the intracellular bacteria *L. monocytogenes*, is a cholesterol-dependent cytolysin (CDC) responsible for a wide array of functions including disruption of the internalization vacuole (54). Further, *M. tuberculosis* has cholesterol uptake machinery, an enzyme system capable of catabolizing sterols for growth and potentially utilizes sterols as a carbon and energy source (55, 63).

It is perhaps unsurprising, given the essential role sterol metabolism plays in their replication, that examples are appearing of pathogens subverting or co-opting the regulation of this system for their own benefit. In 2007, Mackenzie et al. showed that a West Nile virus (WNV) infection of Vero cells induced an upregulation of cholesterol biosynthesis and redistribution of cholesterol resulting in defective IFN-stimulated JAK/STAT signaling. This result was attributed to a disrupted recruitment and activation of the type 1 IFN receptor and IFN signaling proteins and emphasizes the tight integration of IFN and cholesterol regulation in the cell (64).

In summary, evidence increasingly reveals an intimate molecular coupling between IFN signaling and the sterol metabolic network. This underscores the importance of immune-mediated regulation of sterol metabolism as an integral component of the host response to infection.

## Cellular Mechanisms for IFN Regulation of the Sterol Metabolic Network

A prototypic outcome of IFN signaling in the cell is the transcriptional activation or suppression of hundreds of genes. Over the past 5 years, significant progress in characterizing which of these genes contribute to regulation of cholesterol in the cell has been made. In this regard, oxysterol and miRNA-mediated mechanisms have risen to prominence and, in the following sections, we will review what is known about the functions of IFN-elicited CH25H/25-HC and miRNA-mediated sterol regulation. We will then conclude by considering mechanisms of cholesterol regulation by the “conventional” ISG Viperin and the IFITM protein family.

## IFN-Induced 25-Hydroxycholesterol in Infection and Immunity

While cholesterol is a critical component of cell membranes and a precursor of bile acids and steroid hormones, at high concentrations, it may be toxic to the cell. Intracellular cholesterol homeostasis is, therefore, stringently controlled by tightly coupled regulatory mechanisms including influx and efflux, esterification, and storage and biosynthesis (65). Oxysterols are oxygenated forms of cholesterol formed directly from cholesterol (or oxysterols derived from cholesterol) by enzymatic and non-enzymatic mechanisms (55). Functionally, oxysterols such as 22(R)-hydroxycholesterol and 24(S)-hydroxycholesterol potently bind ligand-activated transcription factors liver X receptor (LXR)-α and/or LXR-β and induce the upregulation of cholesterol homeostasis-related proteins such as ABCA1—responsible for cholesterol efflux from the cell (66, 67). Notably, however, despite its identification over 50 years ago and an early demonstration of potent sterol biosynthesis regulatory feedback functionality, until recently, physiological roles for 25-hydroxycholesterol (25-HC) have proven elusive (68, 69).

25-HC binds the INSIG protein in the ER and, in doing so, prevents SREBP2 transport to the golgi/nucleus and cholesterol biosynthesis [reviewed in Ref. (70)]. It is not, however, a strong activator of the LXRs nor does it play a significant role in systemic cholesterol homeostasis *in vivo* (71). In 2009, independent studies showed that CH25H, the enzyme responsible for 25-HC synthesis, is transcriptionally upregulated in macrophages following treatment with a TLR agonist (72, 73). Park and Scott (74) then showed that type I IFNs are also capable of upregulating CH25H (74). While this evidence supported the notion that 25-HC may play a role in immunity, in fact, studies had been emerging for decades implicating 25-HC in the immune response to infection. In 1986, Kournikakis et al. demonstrated that 25-HC can suppress antibody-dependent cell cytotoxicity, and in 1998, Moog et al. showed 25-HC (but not cholesterol) can inhibit HIV *in vitro* (75, 76). Over the next decade, several groups independently investigated the effects of 25-HC on HCV subgenomic replicon replication and found the oxysterol inhibited this process (77–80). Notably, the effects of 25-HC are not restricted to viruses, and in 2006, Howe and Heinzen described a partial inhibition of the bacteria *Coxiella burnetii* following treatment of Vero cells with this oxysterol (81).

## Broad Antiviral Functionality of 25-HC

Interest in the regulation and functions of 25-HC (and its derivatives) has increased dramatically in recent years [reviewed in Ref. (82)]. In 2012, Gold et al. demonstrated that ATF3 directly suppresses the transcription of *CH25H* and the production of 25-HC. They further showed that a deletion of ATF3 in APOE^−/−^ mice results in enhanced aortic 25-HC expression and foam cell development (83). In 2013, Blanc et al. showed that 25-HC is the only oxysterol synthesized (and secreted) in significant quantities by murine macrophages after IFN activation and demonstrated that the transcription of *CH25H* is directly regulated by IFN through the binding of STAT1 to its promoter. These studies also showed that physiological levels of 25-HC have a broad antiviral functionality mediated, in the case of cytomegalovirus (CMV), post-entry *via* regulation of the sterol biosynthesis pathway. Data presented by Blanc et al. supported an important role for the prenylation side-branch of the sterol biosynthesis pathway, rather than cholesterol, in mediating antiviral effects against CMV (52). At the same time, Liu et al. (84), using a molecular screening approach, also identified CH25H as an important IFN-stimulated gene and demonstrated a broad antiviral functionality for 25-HC (84). Contrary to the CMV-related work of Blanc et al., however, Liu et al. found this effect was mediated *via* an inhibition of pathogen [vesicular stomatitis virus (VSV) and (HIV)] entry to the cell. The distinct modes of 25-HC action described likely reflect differences between the cell/virus systems examined. Liu et al. further showed that CH25H^−/−^ mice are more susceptible to MHV-68 infection and the therapeutic administration of 25-HC to humanized mice suppressed HIV-induced T cell depletion (84). Together, these studies identified a significant new role for 25-HC as an effector in the immune response to infection and, since 2013, several independent studies have described further roles for 25-HC in this context. In 2014, Roulin et al. showed that 25-HC suppresses picornavirus infections by displacing cholesterol binding to the oxysterol sterol-binding protein (OSBP1). In doing so, 25-HC disrupts a cholesterol-phosphatidylinositol 4-phosphate counter-current essential for formation of the replication organelle at ER–Golgi membrane contact sites (85). Building on early studies investigating sterol pathway regulation, in 2015, Lu et al. showed that, alongside its ability to inhibit SREBP2 migration to the nucleus, IFN-elicited 25-HC induces a rapid proteosomal degradation of HMGCR in macrophages (6, 86). Work has also shown that 25-HC can inhibit a wide range of unrelated enveloped and non-enveloped viruses including poliovirus, Hepatitis B and C viruses, human papillomavirus (HPV-16), human rotavirus, encephalomyocarditis virus, and SFTS virus (87–92). Recent studies have also revealed more detail regarding the regulation of CH25H. Mboko et al. (93) showed that CH25H expression in mice is, at least partly, dependent on IRF1 and Xiang et al. (94) demonstrated an IFN-independent induction of CH25H in hepatocytes in response to viral infection (93, 94). In this regard, evidence to-date suggests that CH25H gene expression is regulated in a cell-specific manner and it cannot be considered a prototypic ISG. While the majority of studies have broadly focused on the ability of 25-HC to suppress infection *via* regulation of lipid metabolism, data from Shibata et al. (5) suggest that it may also achieve this *via* a specific activation of the GCN2/eIF2α/ATF4 branch of the integrated stress response (ISR) (5).

Notably, recent studies describe direct interactions of both CH25H and 25-HC with gene products of the microorganism. Chen et al. (95) describe a direct interaction between CH25H and NS5A of HCV leading to an inhibition of NS5A dimerization and inhibition of HCV replication (95). More recently, Ren et al. (96) describe an INSIG homolog with predicted 25-HC-binding capacity in the bacterium *Mycobacterium vanbaalenii* (96). What physiological role this would play, however, remains unclear.

In summary, it is now accepted that 25-HC is an important component of the IFN-induced response to infection and a range of studies have identified divergent mechanisms for the inhibition of entry, replication, and exit from the cell.

## 25-HC as an Inflammatory Mediator

Recent evidence has emerged supporting a role for 25-HC as a pro- and/or anti-inflammatory mediator. In 2010, it was demonstrated that 25-HC has the capacity to suppress CCR7 expression and thus impair DC migration (97). Wang et al. subsequently described a 25-HC-elicited RIG-I-dependent induction of IL-8 and Raccosta et al. showed that 25-HC can bind CXCR2 (98, 99). In 2014, Data from Reboldi et al. showed that in macrophages, through its ability to antagonize SREBP, 25-HC can reduce IL-1β expression and inflammasome activation. They further demonstrated that CH25H^−/−^ mice are more sensitive to septic shock and have an enhanced ability to suppress *L. monocytogenes* infection (100). In contrast, Gold et al. (101) describe 25-HC as an amplifier of inflammation, showing a reduction in pro-inflammatory gene expression in poly I:C treated CH25H^−/−^ macrophages and decreased inflammatory pathology in the lungs of Influenza virus-infected mice (101). Further evidence of a pro-inflammatory role for 25-HC has very recently emerged from Jang et al. (102) who describe a role for the oxysterol as an endogenous signal for NLRP3/inflammasome activation during cerebral inflammation (102). At present, therefore, evidence would appear to support multiple roles for 25-HC in the regulation of inflammation.

An important consideration in the analysis and interpretation of 25-HC-related data is the concentration of exogenous oxysterol utilized *in vitro*. Others, and ourselves, have demonstrated that nanomolar concentrations of 25-HC elicit profound effects in primary macrophages, e.g., Ref. (52, 100). In the literature, however, functional roles for 25-HC have been defined after treatment of cells with considerably higher concentrations (e.g., 10–100µM)—for instance (99, 102). Caution should be exercised when interpreting data from experiments utilizing arguably supraphysiological concentrations of the oxysterol. In this regard, more work—in particular *in vivo*—is required to characterize the specific concentrations, circumstances, locations, and times at which pro- or anti-inflammatory effects are observed during infection.

## CH25H and Acquired Immune Responses

In 2009, Bauman et al. described a role for 25-HC in the direct repression of B cell proliferation and immunoglobulin class switching (72). Oxysterols are often subject to consecutive modifications in order that a functional effector molecule can be synthesized, and in 2011, two groups identified 7α,25-HC—generated *via* the hydroxylation of 25-HC by CYP7B1—as a ligand for the receptor EBI2 (71, 82, 103, 104). While studies of 25-HC have broadly focused on its production and function in macrophages, 7α,25-HC is primarily synthesized in radiation resistant stromal cells (105). The 7α,25-HC receptor EBI2 is expressed throughout the immune system and, to-date, has been shown to play a critical role in B lymphocyte and dendritic cell biology. In B lymphocytes, EBI2 binding of 7α,25-HC, induces a series of temporally regulated migratory events. B cells first move to the outer follicles of lymphoid tissues, then migrate to the T cell margin, and finally move to interfollicular regions before EBI2 is downregulated and germinal centers form (82, 106). Ultimately, binding of 7α,25-HC to EBI2 and subsequent B cell repositioning events are crucial to antibody responses and CH25H^−/−^ mice have reduced IgG and IgM responses to T cell-dependent antigens (107, 108). In dendritic cells, EBI2 and 7α,25-HC are also crucial and determine cellular migration/location and ability of these cells to support CD4 and B cells responses to blood borne antigens (82, 109).

Importantly, roles for 25-HC and downstream metabolites in the regulation of T lymphocyte responses are emerging. In 2014, data from Chalmin et al. suggested that 7α,25-HC may direct the migration of activated T cells into the CNS in a model of autoimmune encephalomyelitis. Further, a very recent study from Li et al. (110) has described a role for 25-HC in T helper cell development. Specifically, through an interaction with T lymphocyte EBI2, 7α,25-HC functions to orientate T cells at the interface of the follicle and the T cell zone. In doing so, it promotes T_FH_ cell differentiation by facilitating interactions first between the lymphocytes and ICOSL^HI^ CD25^+^ dendritic cells and subsequently between lymphocytes and B cells (110).

Evidence is rapidly accumulating that multiple complementary mechanisms are responsible for the molecular coupling of IFN to sterol metabolism. In this context, Singaravelu et al. (111) recently described an ability of 25-HC to induce the expression miR-185 and, in doing so, regulate host lipid metabolism pathways critical to HCV replication (111). This finding will be discussed in more detail later.

## How Does 25-HC Suppress Infection?

For almost four decades, a physiological role for 25-HC remained elusive, however, multiple lines of evidence now show that the direct induction of *CH25H* expression and 25-HC synthesis by IFN is a fundamentally important feature of immune responses to infection. A key unanswered question is: *what are the mechanisms by which 25-HC can suppress infection?*

Studies to-date have primarily utilized 25-HC as a research tool to study the functional role of cholesterol homeostasis and its effects on membrane composition, vesicular trafficking, and isoprenylation. The addition of exogenous side-chain oxysterols such as 25-HC to cells is known to elicit trafficking of cholesterol from the membrane to the ER—an event that may perturb membrane architecture and the orientation and composition of, for example, lipid rafts (112). *Via* an interaction with OSBP1, 25-HC is also known to disrupt a cholesterol-phosphatidylinositol 4-phosphate “counter-current” required for ER to golgi cholesterol transport and Rhinovirus replication (85). In 2013, Liu et al. found 25-HC-inhibited membrane fusion and cellular infections by HIV, Ebola, and HCV (84). In contrast, Blanc et al. (52) found that 25-HC had a minimal effect on MCMV entry and, by utilizing a mathematical model, estimated that entry-related effects of this oxysterol account for only 10% of its overall antiviral activity for this virus. Data from Blanc et al. (52) support the view that 25-HC primarily exerts its effects by limiting mevalonate–sterol biosynthesis pathway flux. In particular, flux associated with the isoprenoid branch is responsible for protein prenylation (52). Prenylation refers to the posttranslational modification of proteins by the covalent addition of farnesyl (C_15_) or geranylgeranyl (C_20_) to conserved amino acid motifs and is key to protein–membrane interactions/intracellular localization of, for example, the Rab proteins. The Rab GTPase superfamily has more than 20 members playing essential roles in vesicle trafficking and protein localization in the cell. Prenylation is the key to this function as it allows attachment of the protein to the lipid bilayer. An ability to inhibit the prenylation and, therefore, the function of Rab GTPases may allow 25-HC to influence a wide range of pathogens that depend on or utilize this family of proteins. For example, Rab11 is key to the recycling endosome pathway in cells and plays a critical role in the assembly of multiple negative strand RNA viruses such as respiratory syncitial virus, Influenza A, and Sendai virus (113–115). Notably, the direct prenylation of pathogen proteins is also an important event in some bacterial infections. For example, the PelH and AnkB proteins of *Legionella pneumophila* are known to require farnesylation, while SifA of *Salmonella typhimurium* requires geranylgeranylation for membrane association (116–118). The effects of 25-HC on these bacteria have yet to be characterized. Given the complexity of the Rab superfamily and the differential dependency of a range of pathogens on its functions, more work is required to determine whether 25-HC effects are mediated *via* this route.

An intriguing possibility is that microorganisms may exploit the disruption of Rab prenylation by 25-HC. Rab5a contributes to lysosomal degradation of *L. monocytogenes* in macrophages and a disruption of its geranylgeranylation in this context may prove advantageous to the bacterium. In this regard, data show that the growth of *L. monocytogenes* is greater in WT macrophages when compared to their CH25H^−/−^ counterparts (100).

While several studies have identified an important role for 25-HC *in vivo*, questions remain regarding: where and when 25-HC is synthesized after infection, how 25-HC synthesis is regulated in different anatomical locations, functional *in vivo* intra- and extracellular concentrations, half-life in the tissues and circulation and therapeutic potential (84, 108). In 2014, Ikegami et al. analyzed oxysterol concentrations in serum of patients with chronic HCV infection and found 25-HC levels 44% greater than those in the controls. Notably, 25-HC levels *decreased* significantly in these HCV-infected patients after they had received PEGylated IFN and Ribavirin therapy for a period of 3 months (119).

The presence of INSIG homologs with potentially conserved 25-HC-binding capacity in bacteria and yeast raises the intriguing possibility that the oxysterol can directly influence these organisms. An incomplete understanding of sterol metabolism in these organisms and, in some cases, an absence of SREBP, SCAP, or HMGCR homologs makes progress in this field challenging at present (96).

## IFN-Induced miRNA Regulation of Sterol Metabolism in Infection and Immunity

miRNA are small (20–25 nt) RNA encoded in introns, exons, and intergenic regions of the mammalian genome and are typically co-expressed with a protein-coding or non-coding primary transcript. miRNA function to regulate gene expression *via* imperfect base-pairing to the 3′UTR of mRNA which results in the targeting of the transcript for degradation and/or an inhibition of translation. A key functional characteristic of miRNA is that they can target and regulate the expression of multiple transcripts in the cell. Since the discovery that miRNA, in particular miR-33, function to regulate cholesterol homeostasis, interest in this area has grown dramatically and more than 20 miRNAs are now known to directly target the sterol metabolic network [reviewed in Ref. (120)]. Notably, a small number of sterol-associated miRNA have been shown to be IFN regulated and a subset of these also contribute to the immune response to infection. Here, we will review what is known about these IFN-regulated sterol regulatory miRNA and discuss the mechanisms employed to inhibit pathogens.

## Sterol Pathway Targeting by miR-342-5P Generates Broad Antiviral Immunity

miR-342 is encoded in an intron of the Ena-vasodilator-stimulated phosphoprotein gene (*EVL*) in the mouse or Ena-Vasp-Like (*EVL*) gene in the human and co-transcribed with this transcript. Transcription of miR-342 can be induced by all-trans retinoic acid or IFN and suppressed by CpG island methylation upstream of *EVL*. Processing of the primary transcript results in the production of a pre-miRNA hairpin encoding two functional miRNA—miR-342-3p and miR-342-5p (7, 121–124). *In vivo*, the *EVL* transcript is primarily expressed in cells of the immune and nervous systems, however, a systematic tissue and cell-type analysis of miR-342 expression has yet to be undertaken (125). In macrophages, miR-342 has been identified as a PU.1-regulated miRNA contributing to myeloid differentiation and miR-342-5p shown to be a pro-inflammatory mediator capable of enhancing miR-155 expression (123, 124). miR-342-5p has recently been implicated in the regulation of SREBP1 and SREBP2 in a cancer cell line; however, biological roles and precise mechanisms for the miRNA in relation to sterol biosynthesis and the immune response were not addressed (126). In this regard, we recently demonstrated that, in BMDM, miR-342-5p is directly regulated by IFN *via* IRF1 (7). We further showed that miR-342-5p directly targets the master transcriptional regulator of the pathway SREBP2, multiple members of the sterol biosynthesis pathway including (*IDI1* and *SC4MOL*) and can reduce miR-33 abundance in the cell (7). In doing so, miR-342-5p contributes to IFN-induced suppression of the sterol metabolic network—reducing the abundance of both sterol pathway metabolic intermediates and total cholesterol in macrophages. Notably, IFN-induced miR-342-5p suppression of the sterol metabolic network enables this miRNA to inhibit the replication of unrelated viruses including Influenza A and HSV1 (7). This study, in conjunction with our previous analysis of the antiviral effects 25-HC, highlights the complex, temporally coordinated, and redundant molecular circuitry utilized by the cell to regulate the sterol metabolic network during infection.

**Figure 2:**
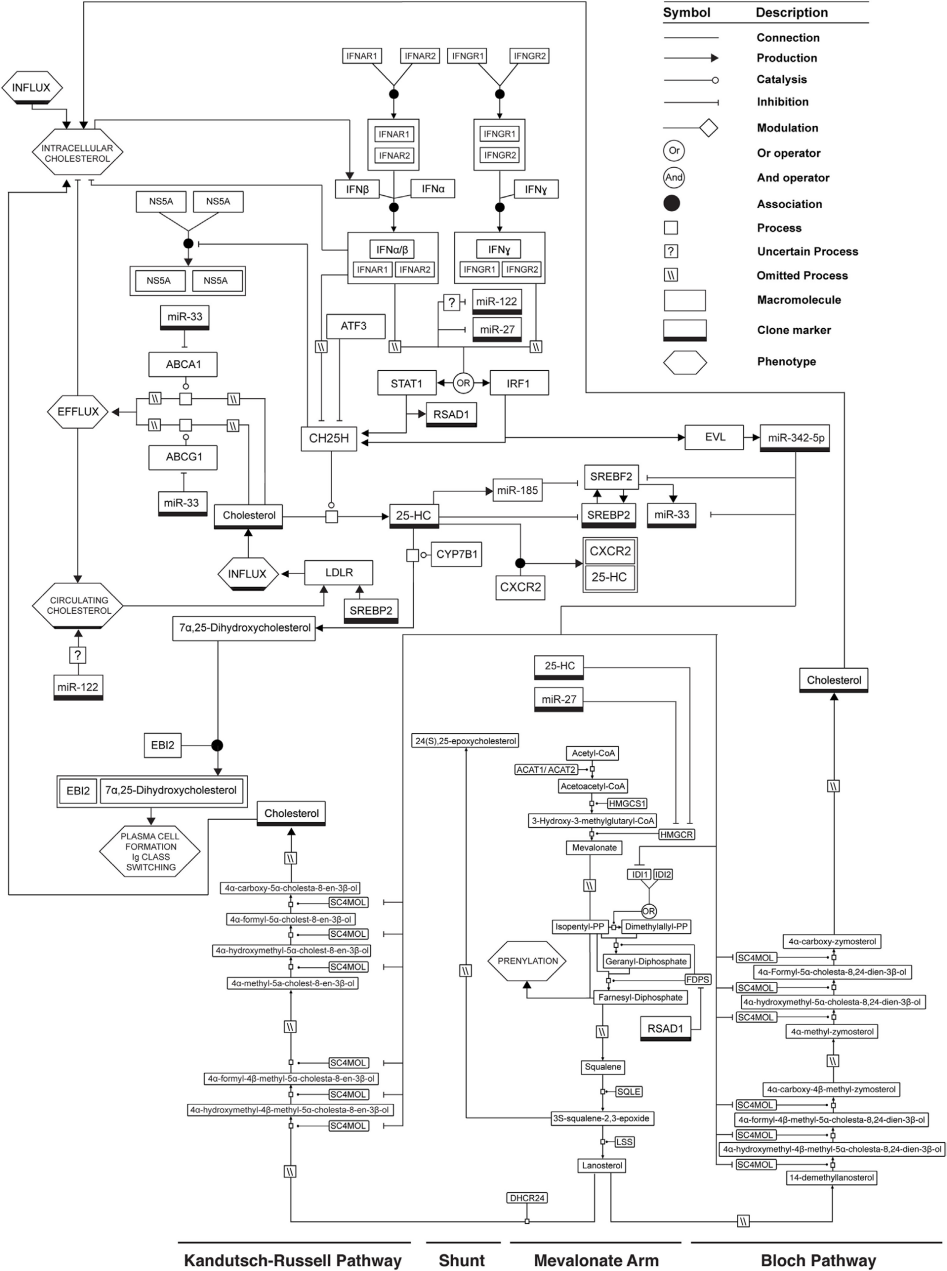
**Mechanisms by which IFN can regulate the sterol metabolic network**. See legend for glyph notation.

A summary of the molecular circuitry underlying the regulation of sterol metabolism by IFN is presented in Figure 2. In murine BMDM, *CH25H* mRNA expression is regulated by STAT1 and increases dramatically in the first half hour after activation of cells by IFN (52). In contrast, miR-342-5p expression increases from 2 to 3 h after IFN-γ activation of BMDM and is regulated by IRF1. Data from others and ourselves suggest, therefore, that a sequential IFN-elicited regulation of the sterol metabolic network exists in which 25-HC provides a rapid mechanism for decreasing sterol biosynthesis. It does this *via* an immediate proteosomal degradation of HMGCR and subsequent inhibition of SREBP2 nuclear translocation. This leads to a suppression of viral entry and/or replication *via* an inhibition of sterol pathway flux—an outcome that will also activate STING to further stimulate type I IFN production. Since IFN-stimulated ATF3 swiftly inhibits CH25H transcription and 25-HC is rapidly catabolized, the CH25H response is primarily effective in limiting sterol synthesis for the first few hours of IFN induction. Importantly, however, miR-342-5p then further promotes a longer, sustained fine-tuning of sterol metabolism, and antiviral effects in the cell by targeting *SREBF2* RNA and transcripts encoding select enzymes of the sterol biosynthesis pathway (e.g., *IDI1* and *SC4MOL*). In this role, miR-342-5p complements and reinforces the antiviral functions of the rapidly induced oxysterol 25-HC on sterol biosynthesis.

While *in vitro* data show that an inhibition of endogenous miR-342-5p can reduce the antiviral effects of exogenous IFN by 40–50%, the relative importance of this miRNA—and also 25-HC—*in vivo* are not known. A critical next step, therefore, will be the production of single miR-342-5p and combined miR-342-5p/CH25H KO murine models in which the individual and combined functions of the miRNA and oxysterol can be tested in the context of infection.

## miR-122 Positively Regulates Cholesterol, Facilitates HCV Replication, and is Downregulated by IFN

Arguably, the first miRNA associated with IFN responses to infection and the regulation of cholesterol metabolism was miR-122. miR-122 is a tissue-specific miRNA highly expressed in hepatocytes in which it constitutes around 70% of all miRNA present in the cell. In agreement with this strict tissue-specific expression, we failed to detect miR-122 in resting or IFN-activated bone marrow-derived macrophages (7). In 2005, Jopling et al. (127) demonstrated a direct interaction between miR-122 and the 5′ region of the Hepatitis C genome and showed that this interaction results in the facilitation of viral replication (127). Subsequently, a role for miR-122 in the regulation of lipid metabolism was revealed when the administration of an antisense oligonucleotide (“antagomir”) to mice resulted in a significant reduction in circulating cholesterol levels (128, 129). Further analyses of miR-122 KO animals confirmed an absence of miR-122 results in reduced plasma cholesterol levels; however, it remains unclear what the specific sterol-related targets of miR-122 are and how this miRNA acts to regulate systemic cholesterol levels (130, 131). Notably, in 2007, miR-122 was identified as an IFN-regulated miRNA whose abundance decreased by around 80% in Huh cells treated with IFNβ (132). Pedersen et al. further showed that the transfection of a miR-122 inhibitor into cells could suppress HCV replication with a similar magnitude of regulation to that induced by IFNβ alone (132). This and other findings have led to the development and *in vivo* testing of therapeutic miR-122 inhibitors that show promise for the treatment of chronic HCV infection (133, 134).

In summary, while miR-122 couples IFN to the regulation of sterol metabolism and, by direct interaction with the viral genome, plays a fundamental role in the replication of HCV, it is currently unknown whether the IFN suppression of miR-122 directly influences circulating cholesterol levels and whether this plays a role in modulating host responses to infection.

## miR-185 is Regulated by 25-HC and Inhibits Virus Replication by Targeting Lipid Metabolism

Recent evidence suggests that miR-185 functions to regulate sterol metabolism in the liver during an immune response to infection. In the absence of an infection or IFN treatment, data from hepatocytes show that miR-185 expression is downregulated when cholesterol is depleted *in vitro* and that expression of this miRNA is directly regulated by SREBP1c *via* a single sterol response element in its promoter (135). Multiple studies have further shown that this miRNA can regulate *SR-BI, SREBP1c, SREBP2, HMGCR, and LDLR* transcript abundance (126, 136, 137). Notably, in 2015, Li et al. showed that HCV can upregulate SREBP2 *via* a core protein-mediated suppression of miR-185 (138) while Singaravelu et al. demonstrated that miR-185 expression in hepatocytes is upregulated by 25-HC and restricts HCV replication *via* a repression of cellular lipid uptake and biosynthesis (111). Data suggest, therefore, that miR-185 is antiviral, indirectly upregulated by IFN through 25-HC and exerts its effects (at least in hepatocytes) *via* a suppression of the sterol metabolic network. This mode of regulation was not detected in activated or infected murine macrophages and the general antiviral significance of these observations in alternative cell types has yet to be tested (7). Importantly, however, these data strongly support previous findings demonstrating a membrane-independent antiviral mechanism for 25-HC.

## miR-27 Integration of Immunity and Lipid Metabolism

Over the past decade, functional roles for miR-27 have been investigated in the context of several viral infections. In this regard, significant attention has focused on the ability of Herpesvirus saimiri and murine CMV to induce a reduction in miR-27 abundance and the virus-related mechanisms mediating this reduction are now relatively well characterized (139–142). Notably, the functional consequences and benefit to the Herpesviruses of this reduction are incompletely understood with studies focusing on a suppression of miR-27-inducing constitutive T cell activation (Herpesvirus saimiri) or the suggestion that this event enhances IL-10 production during MCMV infection (142). Since 2013, several studies have described a miR-27 regulation of lipid (including sterol) metabolism. In 2013, Vickers et al. described a miR-27-mediated reduction in *HMGCR* abundance and a sensitivity of miR-27 to lipid levels *in vivo* (143). Also at this time, Shirasaki et al. described a HCV (but not IFN) induction of miR-27a in hepatocytes and a repression of *ABCA1, SREBP1*, and *SREBP2* by the miRNA. They further showed that an inhibition of miR-27 increased cellular lipids/viral replication and an over-expression of the miRNA resulted in a reduction in viral infectivity and enhanced IFN signaling (144). In 2014, Singaravelu et al. showed HCV induction of miR-27 is accompanied by the formation of large, abundant lipid droplets in hepatocytes. Zhang et al. further demonstrated this miRNA directly targets *ABCA1, LPL*, and *ACAT1*, and, in doing so, reduces cholesterol efflux/uptake and regulates the balance of free versus esterified cholesterol in THP1 cells (145). Notably, recent work from Zheng et al. (146) describes a type 1 IFN downregulation of miR-27 in macrophages resulting in enhanced SIGLEC1/TRIM27 expression. As a consequence, IFN signaling was suppressed and VSV replication enhanced (146). Taken together, the above studies suggest that miR-27 couples infection-induced IFN responses to the regulation of sterol metabolism. Importantly, however, the significance of miR-27 and the relative importance of its sterol-regulatory effects in the context of specific infections and cell types are, incompletely understood. In this regard, we and others have demonstrated that MCMV is dependent on the sterol metabolic network for its replication. It may be hypothesized, therefore, that a suppression of miR-27 functions to upregulate the sterol metabolic network and, in doing so, enhances viral replication.

Alongside the examples discussed above, several other miRNAs hold promise as IFN-regulated modulators of the sterol metabolic network. Others, and ourselves, have demonstrated an IFN-elicited downregulation of miR-33 (7, 147). Recent work from Lai and colleagues demonstrates that miR-33 promotes pro-inflammatory signaling *via* an ABCA1/ABCG1 augmentation of lipid raft microdomains (147). IFN-mediated downregulation of this miRNA, therefore, may serve to suppress the ongoing inflammatory response. Both viral infection and IFNγ can induce the expression of miR-19b—known to target *ABCA1* (148, 149). Further, type 1 IFN (and hepatitis B virus) suppress the expression of miR-145—a miRNA known to target *ABCA1* and HPV and play a role in the progression of atherosclerosis (150–154).

In summary, through their ability to simultaneously regulate multiple genes and propensity for fine-tuning rather than the induction of dramatic alterations in RNA expression, miRNA are ideally suited to the task of coordinating protective functions of the sterol metabolic network (on which the cell depends). Notably, work to-date supports cell- or tissue-specific expression of IFN-regulated miRNAs. In this regard, a great deal is still unknown about where and when IFN-regulated miRNA are expressed *in vivo*, how they are regulated by IFN and what their targets are in particular cell types in distinct species. In this regard, knockout miRNA models remain relatively scarce. There is a pressing need, therefore, for the development of new models to enhance our understanding of sterol regulatory miRNA and the roles they play in host protection against infection.

## ISG Regulation of the Sterol Metabolic Network During Infection

While this review has focused on IFN-elicited oxysterol and miRNA-related mechanisms, several “conventional” ISG, integral to the cellular response to infection, also elicit their effects *via* the sterol metabolic network.

## Viperin

Work characterizing the IFN-regulated gene Viperin (virus inhibitory protein, endoplasmic reticulum-associated, IFN-inducible, or *RSAD2*) has demonstrated that this protein can inhibit both RNA and DNA viruses (155–158). Viperin can decrease HCMV late gene expression, block the release of Influenza A and HIV-1 particles from the cell, and limit the replication of HCV, dengue virus, and WNV [reviewed in Ref. (159)]. Importantly, *in vitro* data show that Viperin inhibits Influenza A budding by disrupting cell membrane lipid raft integrity and increasing membrane fluidity. A key feature of this mechanism is the binding of Viperin to the sterol pathway enzyme farnesyl diphosphate synthase (FDPS)—an enzyme integral to sterol biosynthesis and the processes of farnesylation and geranylgeranylation (156, 158). Together, these observations suggest Viperin functions to inhibit Influenza A release *via* regulation of the sterol metabolic network; however, a precise mechanism has yet to be determined. Unlike Influenza A and other viruses such as HIV-1 and Ebola, HCV does not bud from lipid rafts. HCV does, however, have an intimate relationship with cellular lipid metabolism—in particular, a dependency on lipid droplets. It has been suggested, therefore, that Viperin may inhibit HCV replication by altering the lipid composition of these droplets *via* its interaction with FDPS. This has not, however, been confirmed (160). Very recently, a TLR4/IRF3-dependent Viperin-induced reduction in membrane cholesterol and sphingomyelin was found to be key to the inhibition of Rabies virus replication in RAW264.7 cells (161). Taken together, the above studies highlight the importance of Viperin as a very early IFN-induced antiviral protein. While our mechanistic understanding is incomplete, it is notable that Viperin exerts at least some of its effects *via* the specific targeting of a key enzyme in the sterol metabolic network and a profound alteration of cellular membrane composition. Further work is required to confirm a conclusive link between these two observations and investigate mechanisms by which pathogens can exploit this protein for their own benefit (162).

## IFITM3

While the IFN-inducible transmembrane (IFITM) proteins were first described some 20 years ago, their antiviral properties remained unknown until 2009 when Brass et al. demonstrated a functional role in cellular resistance to Influenza A, WNV, and Dengue virus (163). Since 2009, a plethora of studies have demonstrated the importance of IFITM proteins in suppressing virus-related morbidity and mortality and have characterized roles for the IFITM proteins in responses to a range of enveloped and non-enveloped viruses [reviewed in Ref. (164)]. Much of this work has focused on the ability of IFITM proteins to inhibit viral entry and/or the very early stages of viral replication. In this regard, in 2013, Amini-Bavil-Olyaee et al. demonstrated that IFITM1, 2, and 3 interact with vesicle-associated membrane protein A (VAPA) (165). VAPA is a highly conserved protein, generally found in the ER and, importantly, known to play a role in cholesterol homeostasis *via* its interaction with OSBP. Under normal circumstances, OSBP is found in the cytoplasm where it serves as a cholesterol sensor and, together with VAPA, functions to redistribute cholesterol from the ER to other organelles in the cell. Amini-Bavil-Olyaee et al. (165) found that an IFITM-mediated disruption of the VAPA–OSBP interaction results in cholesterol levels increasing dramatically in late-endosomal compartments. They attributed a block in VSV release into the cytosol to this alteration in membrane composition (165). Notably, subsequent studies suggest an IFITM-mediated regulation of SNAREs and/or regulation of protein lateral mobility may explain the ability of these proteins to inhibit IAV entry to the cell (166). Interestingly, Munoz-Moreno et al., very recently, described a role for IFITM2 in protecting Vero cells against African Swine Fever Infection—a DNA virus (167). In agreement with previous work, they also described an IFITM-associated accumulation of cholesterol in cells, however, it remains unclear whether an IFN-induced IFITM-mediated regulation of the sterol metabolic network plays a direct role in the antiviral functions of this family of proteins.

## Concluding Remarks and Future Perspectives

Three decades after Cantell and colleagues speculated that IFN regulates sterol metabolism, the first evidence for a molecular coupling of IFN to the sterol metabolic network was provided by a systems biology investigation of macrophage responses to infection (4, 13). Given the wealth of clinical and molecular data now available, it is clear that an IFN-mediated reshaping of the sterol metabolic network is an integral, core component of the immune response to infection. The functional outcomes of this event are, however, only beginning to emerge for the wide array of immune-related cells and tissues in the body and are likely to be complex and context dependent. For example, in secondary lymphoid organs, 25-HC is indispensible as an intermediate metabolite crucial to B cell, T cell, and DC migration and antibody class switching. At a cell-intrinsic level, however, this oxysterol can also inhibit viral entry and replication. The latter occurs *via* the regulation of sterol biosynthesis through SREBP2 and HMGCR in macrophages. A key question arising from work to-date is: *how does the sterol metabolic network influence immunity?* Studies now show that the molecular coupling between IFN and sterol metabolism is bidirectional. In this regard, the recent work of York et al. (33) is fascinating as it demonstrates the influence of sterol metabolic flux on antiviral IFN signaling (33). Similarly, Reboldi et al. recently showed that the transcription factor SREBP2, whose function is tightly coupled to cholesterol homeostasis, functions to regulate inflammatory responses to infection and it has been demonstrated that a cholesterol loading of macrophages leads to a reduction in miR-342-5p abundance (33, 100, 168). In this context, relatively little is known about how the microbiome and diet, in particular, cholesterol intake, affect IFN responses to infection and this is an important question for the future.

Work to-date emphasizes the complexity of the molecular circuitry governing the regulation of sterol metabolism by IFN and vice versa. Given IFN directly or indirectly regulates the expression of several hundred genes and the inherent complexity of the sterol metabolic network; it is likely that new bidirectional regulatory mechanisms will continue to appear. For example, we have shown that alongside the posttranscriptional and posttranslational effects of miR-342-5p and 25-HC, IFN can also repress SREBF2 transcription. The mechanism for this is unclear, however, epigenetic modifications may play a critical role and this will be important area to pursue in the future.

Given the many recent advances in our understanding of the role sterol metabolism plays in immunity to infection, how can we translate our new knowledge to clinical applications? While changes in systemic cholesterol levels may be of diagnostic value, the therapeutic targeting of host metabolic pathways for anti-infective treatment represents the most exciting application of our knowledge to-date. While statins are a widely utilized, clinically approved, therapy for regulating sterol metabolism and can inhibit a range of pathogens *in vitro, in vivo* utility in the context of infectious diseases remains inconclusive. The emergence of new pathogens and threat of antibiotic resistance means it is imperative that we develop new methods for treating infectious diseases. While studies have explored oxysterol and miRNA inhibitor regulation of sterol metabolism in a preclinical and clinical context, legitimate concerns have been raised about the pharmacokinetics and potential side-effects of both. For example, the miR-122 inhibitor Miraversin can be effectively delivered *in vivo* and substantially reduces HCV replication in a Chimpanzee model. Importantly, however, Miraversin administration is typically accompanied by an increase in circulating cholesterol leading to concerns that the cardiovascular health of recipients may be affected. Further, while miR-342-5p regulates sterol biosynthesis and, in doing so, can suppress viral replication it also targets AKT1 and, as a result, can promote inflammation (124). An important objective, therefore, will be to identify the specific mechanisms by which IFN-induced regulators of the sterol metabolic network function to suppress pathogen replication and specifically target these molecules. In doing so, undesired off-target effects will be reduced. In this context, several groups have already explored prenylation as a viable therapeutic target. Prenylation inhibitors are available as an oral medication and show promise in the treatment of, for example, HDV (169).

In conclusion, the sterol metabolic network has now moved center-stage in the context of IFN responses to infection and is increasingly recognized as a fundamentally important immune-metabolomic system holding great promise in the next decades as target for diagnostic and therapeutic intervention.

## Author Contributions

KAR wrote first draft of manuscript. KAR and PG edited and revised final manuscript.

## Conflict of Interest Statement

The authors declare that this document was written in the absence of any commercial or financial relationships that could be construed as a potential conflict of interest.

## Abbreviations

7α,25-HC, 7α,25-dihydroxycholesterol; 25-HC, 25-hydroxycholesterol; CH25H, cholesterol 25-hydroxylase; EBI2, EBV-induced G-protein coupled receptor 2 (also known as GPR183); HCV, hepatitis C virus; HDL, high-density lipoprotein; HIV-1, human immunodeficiency virus 1; HMGCR, 3-hydroxy-3-methylglutaryl-CoA reductase; HSV1, herpes simplex virus type 1; IAV, influenza A virus; IFN, interferon; IRF, interferon regulatory factor; LDL, low-density lipoprotein; LXR, liver X receptor; MCMV, murine cytomegalovirus; MHV-68, murine gammaherpesvirus 68; SREBP2, sterol regulatory-binding protein 2; SREBF2, sterol regulatory-binding transcription factor 2; TLR, toll-like receptor; VSV, vesicular stomatitis virus; VZV, varicella zoster virus; WNV, West Nile virus.
